# A new method for predicting essential proteins based on participation degree in protein complex and subgraph density

**DOI:** 10.1371/journal.pone.0198998

**Published:** 2018-06-12

**Authors:** Xiujuan Lei, Xiaoqin Yang

**Affiliations:** School of Computer Science, Shaanxi Normal University, Xi’an, China; Weizmann Institute of Science, ISRAEL

## Abstract

Essential proteins are crucial to living cells. Identification of essential proteins from protein-protein interaction (PPI) networks can be applied to pathway analysis and function prediction, furthermore, it can contribute to disease diagnosis and drug design. There have been some experimental and computational methods designed to identify essential proteins, however, the prediction precision remains to be improved. In this paper, we propose a new method for identifying essential proteins based on Participation degree of a protein in protein Complexes and Subgraph Density, named as PCSD. In order to test the performance of PCSD, four PPI datasets (DIP, Krogan, MIPS and Gavin) are used to conduct experiments. The experiment results have demonstrated that PCSD achieves a better performance for predicting essential proteins compared with some competing methods including DC, SC, EC, IC, LAC, NC, WDC, PeC, UDoNC, and compared with the most recent method LBCC, PCSD can correctly predict more essential proteins from certain numbers of top ranked proteins on the DIP dataset, which indicates that PCSD is very effective in discovering essential proteins in most case.

## Introduction

Proteins are the products of genes, and they are the vital material and functional units for living organisms. Essential proteins are those proteins which are indispensable for organisms to normally grow and multiply. Thus accurately identifying essential proteins makes important contribution to understanding the key biological processes of an organism at molecular level, which is beneficial to guide disease diagnosis and drug design.

In the previous studies, both experimental and computational approaches have been exploited to detect essential proteins. The experimental approaches for identifying essential proteins, such as single gene knockout [1], RNA interference [2] and conditional knockout [3], all of which are time consuming and expensive. Consequently, a large number of computational approaches are developed to identify essential proteins with the support of large-scale PPI data gained by utilizing high-throughput techniques. Initially, computational approaches mainly focused on the topological properties of biological networks, and there are a series of topological centrality measures following the “centrality-lethality” principle. Among these centrality measures, Degree Centrality (DC) [4], Betweeness Centrality (BC) [5], Closeness Centrality (CC) [6], Eigenvector Centrality (EC) [7], Information Centrality (IC) [8] and Subgraph Centrality (SC) [9] are the classical ones. In addition, some other effective centrality measures, i.e., maximum neighborhood component (MNC) and density of maximum neighborhood component (DMNC) [10], Local Average Connectivity (LAC) [11], Neighborhood Centrality (NC) [12], local interaction density (LID) [13], TP and TP-NC [14] have been also designed to identify essential proteins. CytoNCA [15], a plugin of Cytoscape for centrality analysis and evaluation of biological networks, has been developed to conveniently predict essential proteins. However, all these topological centrality measures ignore the intrinsic biological characteristics of essential proteins and there are a lot of false positives and false negatives in PPI networks, thus the identification accuracies of essential proteins were affected. To overcome these limitations, many researchers attempt to combine network topology and biology information.

Based on the combination of gene expression profiles and PPI data, Li et al. proposed an approach named PeC [16] and Tang et al. proposed a modified one named WDC [17]. By analyzing the correlation between proteins and their domain features, Peng et al. proposed a new prediction method, named UDoNC, by combining the domain features of proteins with their topological properties in PPI network [18]. Peng et al. proposed another method named ION [19] by integrating the orthology with PPI networks, which is based on random walk model. Based on sub-network partition and prioritization by integrating subcellular localization information, Li et al. proposed a new network-based essential protein prediction method, named SPP [20]. Moreover, some researchers exploit protein complexes information to predict essential proteins. For example, Luo et al. proposed LIDC for predicting essential proteins by combing local interaction density with in-degree centrality of complexes [21]. Qin et al. proposed LBCC, which is based on the combination of local density, betweenness centrality (BC) and in-degree centrality of complex (IDC) [22]. Li et al. proposed UC to identify essential proteins by integrating protein complexes with topological features of PPI networks [23]. In addition, to diminish the impacts of inherent false negatives and false positives in PPI data, Li et al. purified the PPI network by integrating gene expressions and subcellular localizations to construct a reliable network [24] [25], and Chen et al. constructed integrated dynamic PPI networks by employing RNA-Seq datasets [26]. There is a detailed introduction about essential proteins discovery methods based on the PPI networks in [27].

In this study, based on the integration of participation degree in protein complexes and subgraph density, a new centrality measure method PCSD is proposed. First of all, refined PPI networks (RPINs) are constructed by applying gene expressions. We calculate the participation degree in complexes for each protein based on the weighted RPINs generated by Edge Clustering Coefficient (*ECC*) and Pearson Correlation Coefficient (*PCC*). We construct a subgraph for each protein, which is compose of the protein as well as its direct (level 1) and indirect (level 2) neighbors, and weight the interactions in the subgraph based on sharing GO annotations (*SG*) and sharing protein complexes (*SC*), then the subgraph density is measured. Finally, a linear combination model is used to integrate two parts of score. The experiment results show that the proposed method PCSD outperforms other existing methods, such as DC, SC, EC, IC, LAC, NC, WDC, PeC, UDoNC, and so on.

The remainder of the paper is organized as follows. Section 2 describes the PCSD algorithm in details. Section 3 presents the computational experiment results and analysis, and Section 4 concludes the paper.

## Methods

### Refined PPI network construction

It is well known that the protein interactions are changing over time, environments and different stages of cell cycle [28], thus the original PPI networks cannot accurately reflect the real protein interactions in cell. In this study, we construct relatively reliable PPI networks by utilizing time-course gene expression data according to three-sigma principle [28]. The three-sigma principle is used to determine the active threshold for each protein based on the characteristics of its expression curve. For a time point, a gene is considered to be expressed if its corresponding gene expression value is greater than or equal to its active threshold. Two proteins should have higher possibility to physically interact with each other if their corresponding genes are both expressed at the same time point [24], in this case, the two proteins are also called as co-expressed protein pairs. We delete those PPIs whose two corresponding proteins are not co-expressed at any time point from original PPI networks. Consequently, a refined PPI network (RPIN) can be constructed.

### Participation degree in protein complexes

In this section, we will analyze the essentiality of proteins in terms of participation degree of proteins in complexes. At first, the RPINs need to be weighted. Previous studies show that both the Edge Clustering Coefficient (*ECC*) and Pearson Correlation Coefficient (*PCC*) are effective ways to weight PPIs [29] [30], which measure the degree of closeness of physical interactions and the strength of co-expression between two proteins, respectively. Therefore, our method PCSD weights RPINs by integrating *ECC* (see Eq (1)) and *PCC* (see Eq (2)). The Edge Clustering Coefficient (*ECC*) between protein *v*_*i*_ and *v*_*j*_ is defined as [31]:

$${ECC}_{ij}=\frac{Z_{ij}+1}{min\{d_{i},{\;d}_{j}\}}$$ (1)

where *Z*_*ij*_ is the number of triangles the edge (*v*_*i*_, *v*_*j*_) actually participates in, *d*_*i*_ and *d*_*j*_ denote the degree of protein *v*_*i*_ and *v*_*j*_, respectively. The Pearson Correlation Coefficient (*PCC*) between protein *v*_*i*_ and *v*_*j*_ is defined as:

$${PCC}_{ij}=\frac{\sum_{k=1}^{n}\left(x_{i}-\overset{-}{x})(y_{i}-\overset{-}{y}\right)}{\sqrt{\sum_{k=1}^{n}{\left(x_{i}-\overset{-}{x}\right)}^{2}}\sqrt{\sum_{k=1}^{n}{\left(y_{i}-\overset{-}{y}\right)}^{2}}}$$ (2)

where *x* = {*x*_*1*_, *x*_*2*_,…, *x*_*n*_} and *y* = {*y*_*1*_, *y*_*2*_,…, *y*_*n*_} give the gene expression values of protein *v*_*i*_ and *v*_*j*_ at *n* time points, $\overset{-}{x}$ and $\overset{-}{y}$ represent the mean of gene expression value of *x* and *y*, respectively. The *PCC* values range from -1 to 1, for convenience, this study replaces *PCC*_*ij*_ by (*PCC*_*ij*_+1)/2. By integrating *PCC* and *ECC*, the probability that two proteins interact with each other can be described from the perspective of network topology and gene expression, therefore, the importance of the interaction between protein *v*_*i*_ and *v*_*j*_ is defined as follows:

$$W_{ij}={ECC}_{ij}\times {PCC}_{ij}$$ (3)

And the weighted degree (sum of weights, *SW*) of protein *v*_*i*_ is defined as:

$$SW(v_{i})=\sum_{v_{j}\in N(v_{i})}W_{ij}$$ (4)

where *N*(*v*_*i*_) is the neighbors set of protein *v*_*i*_.

Protein complexes are stable macromolecular assemblies that play a key role in diverse biochemical activities. [23] suggested that it is more possible to be essential for the proteins included in complexes than those not included in any complexes and the proteins appeared in multiple complexes are more inclined to be essential compared with those only appeared in a single complex. In our design, we calculate the participation degree of a protein in complexes to help characterizing the essentiality of proteins. Proteins participating in complexes includes direct participation and indirect participation. If a protein is included in complexes, that is to say, the protein directly participate in complexes. And if a protein isn’t included in any complexes, but its some neighbors appear in complexes, in this case, the protein indirectly participate in complexes. Otherwise, the protein doesn’t participate in complexes. The Participation degrees in Complexes (*PC*) of protein *v*_*i*_ is defined as

$$PC\left(v_{i}\right)=\{\begin{array}{ll} \sum_{v_{i}\in C_{i}}SW_{in}\left(v_{i},C_{i}\right), & v_{i}\in V\left(\left|C\right|\right)\\ \sum_{v_{j}\in V\left(\left|C\right|\right)}W_{ij}, & v_{i}\notin V\left(\left|C\right|\right) \end{array}$$ (5)

where *V*(|*C*|) represents all the proteins which are contained in some complexes, *C*_*i*_ represents the protein complexes which contain protein *v*_*i*_ and *SW*_*in*_(*v*_*i*_, *C*_*i*_) denotes weighted degree of protein *v*_*i*_ in the complex *C*_*i*_.

### Subgraph density

In this section, we assess the essentiality of proteins by considering local properties of proteins in a PPI network, and construct a subgraph for each protein within the second order of neighbors. By doing this, the new technique can measure topological information in a larger area. Owing to the small-world property of the majority of biological networks, an index related to higher order neighbors may involve too many nodes, which is not efficient for detecting the essentiality of nodes [26]. Thus, we think that within the second order of neighbors is enough. Previous researches on protein complex detection [32] and essential protein prediction [33] suggest that the performance of the prediction algorithm based on weighted networks is superior to that based on un-weighted networks. Therefore, to calculate the subgraph density, we weight the PPIs between protein pairs in subgraphs by applying GO annotations and protein complexes information. If there are some sharing GO annotations between two interacting proteins, the two proteins have the same function, and the interaction between them becomes strong [30]. We define *SG*_*ij*_ to describe the relationship (see Eq (6)). Similarly, if two interacting proteins are contained in a common complex, the interaction between proteins becomes more reliable. We define *SC*_*ij*_ to describe the relationship (see Eq (7)).

$$SG_{ij}=\{\begin{array}{ll} \frac{{\left|G_{i}\cap G_{j}\right|}^{2}}{\left|G_{i}\left|\times \right|G_{j}\right|} & \left|G_{i}\right|>0\;and\;\left|G_{j}\right|>0\\ 0 & \text{otherwise} \end{array}$$ (6)

$$SC_{ij}=\{\begin{array}{ll} \frac{{\left|C_{i}\cap C_{j}\right|}^{2}}{\left|C_{i}\left|\times \right|C_{j}\right|} & \left|C_{i}\right|>0\;and\;\left|C_{j}\right|>0\\ 0 & \text{otherwise} \end{array}$$ (7)

where |*G*_*i*_| and |*G*_*j*_| denote the number of GO annotations for protein *v*_*i*_ and *v*_*j*_, respectively. |*G*_*i*_ ∩ *G*_*j*_| denotes the number of sharing GO annotations for both protein *v*_*i*_ and protein *v*_*j*_. |*C*_*i*_| and |*C*_*j*_| denote the number of protein complexes containing protein *v*_*i*_ and *v*_*j*_, respectively. |*C*_*i*_ ∩ *C*_*j*_| denotes the number of sharing protein complexes annotating both protein *v*_*i*_ and protein *v*_*j*_. Finally, the Subgraph Density (*SD*) of *v*_*i*_ within its second order of neighbors is defined as follows.

$$SD(v_{i})=\frac{2\times \sum \left({SG}_{ij}+{SC}_{ij}\right)}{N_{s}\times (N_{s}-1)}$$ (8)

where *Ns* denotes the number of the proteins contained in a subgraph.

### Essential protein prediction method PCSD

Our method PCSD can rank all proteins in RPINs according to their computed scores. The final essentiality scores is determined by two components: one is the participation degree in complexes *PC* scores obtained in **2.2** section, the other is the subgraph density *SD* scores obtained in **2.3** section. A linear combination model is used to integrate *PC* and *SD* score. For a given protein *v*_*i*_, its essentiality is evaluated by PCSD(*v*_*i*_):

$$PCSD(v_{i})=\alpha \times PC(v_{i})+(1-\alpha )\times SD(v_{i})$$ (9)

where α is a parameter to adjust the contributions of *PC* and *SD*. When α = 0, only the subgraph density is considered, and when α = 1, only the participation degree in complexes is considered. We will discuss the value of α in detail in Experiments and Results section.

## Results and discussion

### Experimental data

In order to evaluate the performance of proposed method PCSD, we conduct a group of experiments on *Saccharomyces cerevisiae* protein data. Four sets of PPI network data were used, including DIP [34], Krogan [35], MIPS [36], Gavin [37]. DIP PPIs were downloaded from (http://dip.mbi.ucla.edu/dip/). MIPS PPIs were downloaded from (ftp://ftpmips.gsf.de/fungi/Saccharomycetes/CYGD/). The PPIs data of Krogan and Gavin come from BioGRID database version 3.4.142 [38]. All self-interactions and repeated interactions were removed as a data preprocessing of these PPIs. The details of all these four PPIs are presented in Table 1. The known essential proteins data were collected from four different databases: MIPS [39], SGD [40], DEG [41] and SGDP [42]. Gene expression data were obtained from GEO (Gene Expression Omnibus) [43] with accession number GSE3431. It contains 9336 genes at 36 time points in 3 cell metabolism cycles. Proteins with gene expression data cover 96.98% of proteins in the DIP data, 98.88% of proteins in the Krogan data, 97.80% of proteins in the MIPS data and 99.16% of proteins in the Gavin data. The GO data we used in this study are cut-down version of the GO ontologies [44], which is available at (http://www.yeastgenome.org/download-data/curation). 745 protein complexes were collected from four protein complex datasets: CM270 [39], CM425 [45], CYC408 and CYC428 [46] [47], which covered 2167 proteins in total.

**Table 1:** The detail information of the four PPI datasets.

Dataset	Proteins	Interactions	Density	Essential proteins
**DIP**	5093	24743	0.0018	1167
**Krogan**	2674	7075	0.0020	784
**MIPS**	4546	12319	0.0012	1016
**Gavin**	1430	6531	0.0064	617

### Comparison with other methods

**Fig 1:**
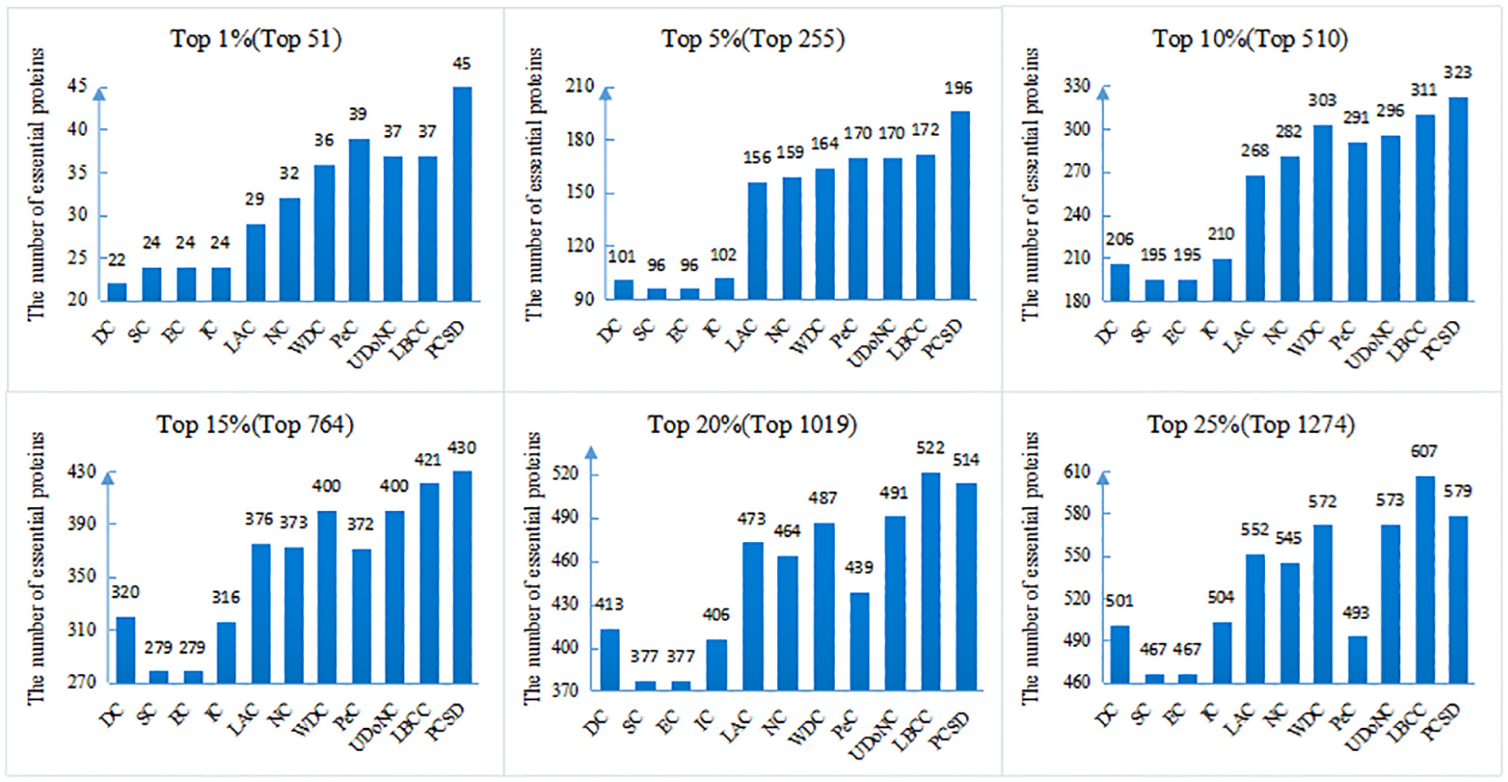
The number of true essential proteins predicted by PCSD and other several methods on DIP dataset.

**Fig 2:**
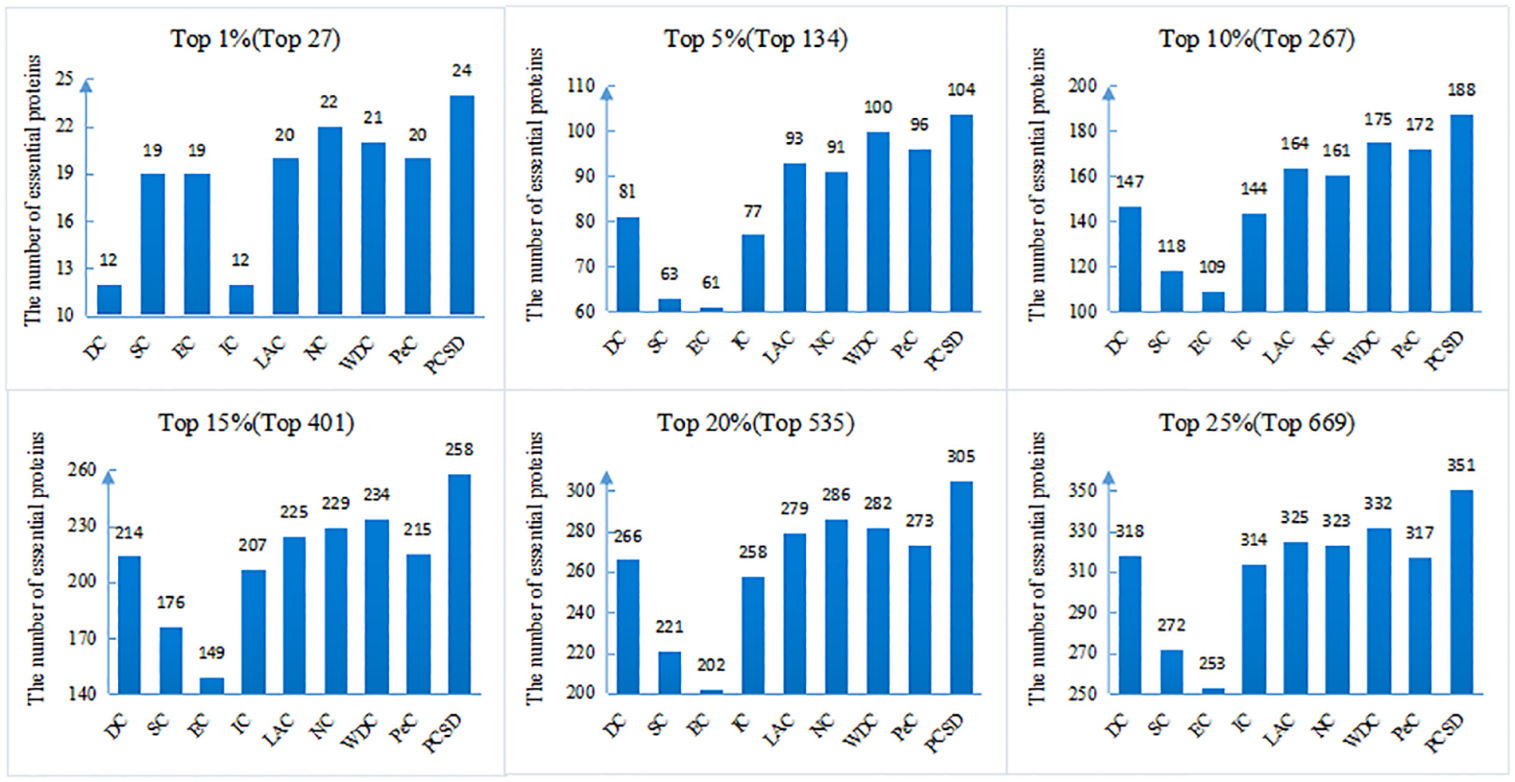
The number of true essential proteins predicted by PCSD and other several methods on Krogan dataset.

**Fig 3:**
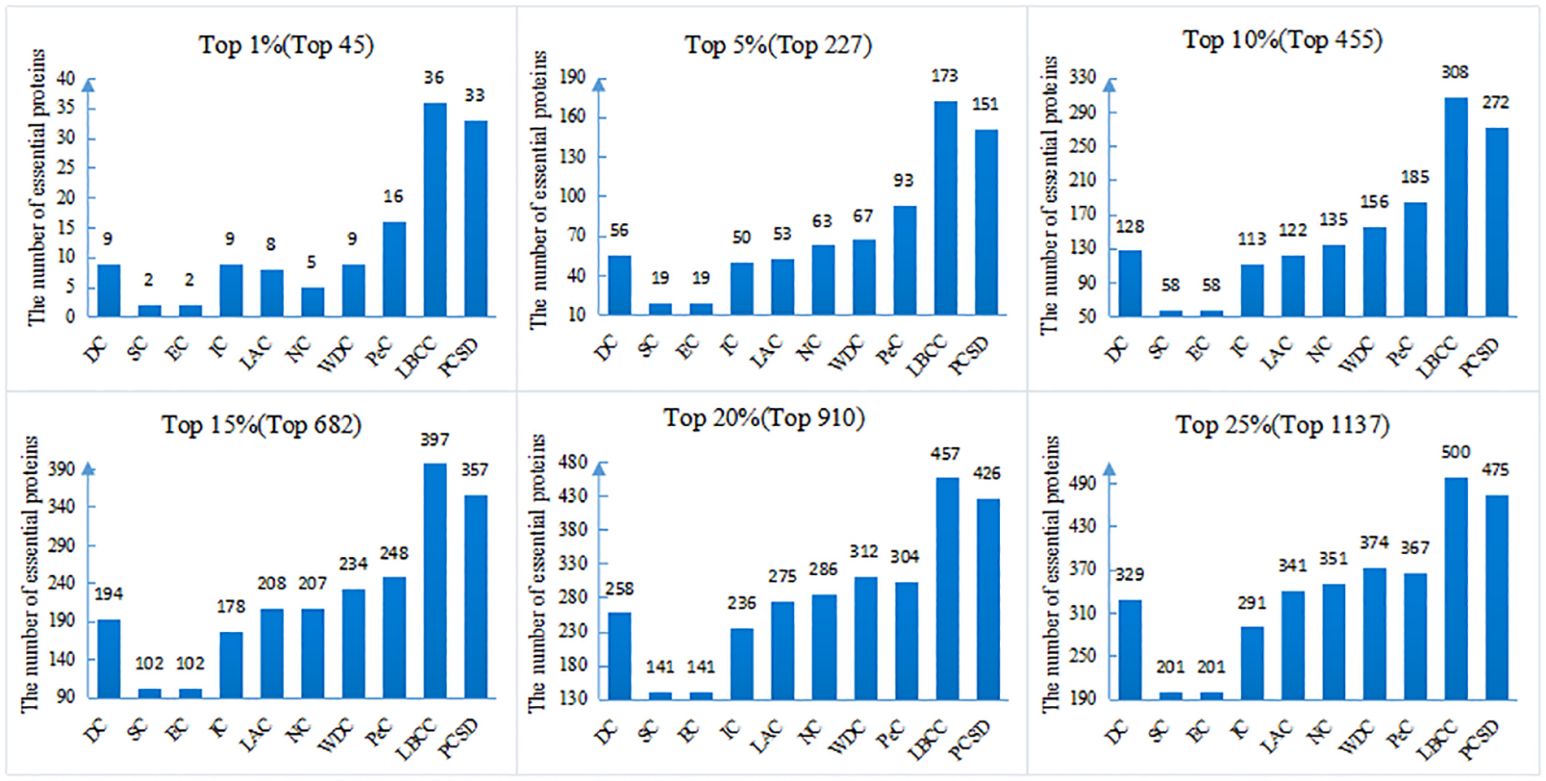
The number of true essential proteins predicted by PCSD and other several methods on MIPS dataset.

**Fig 4:**
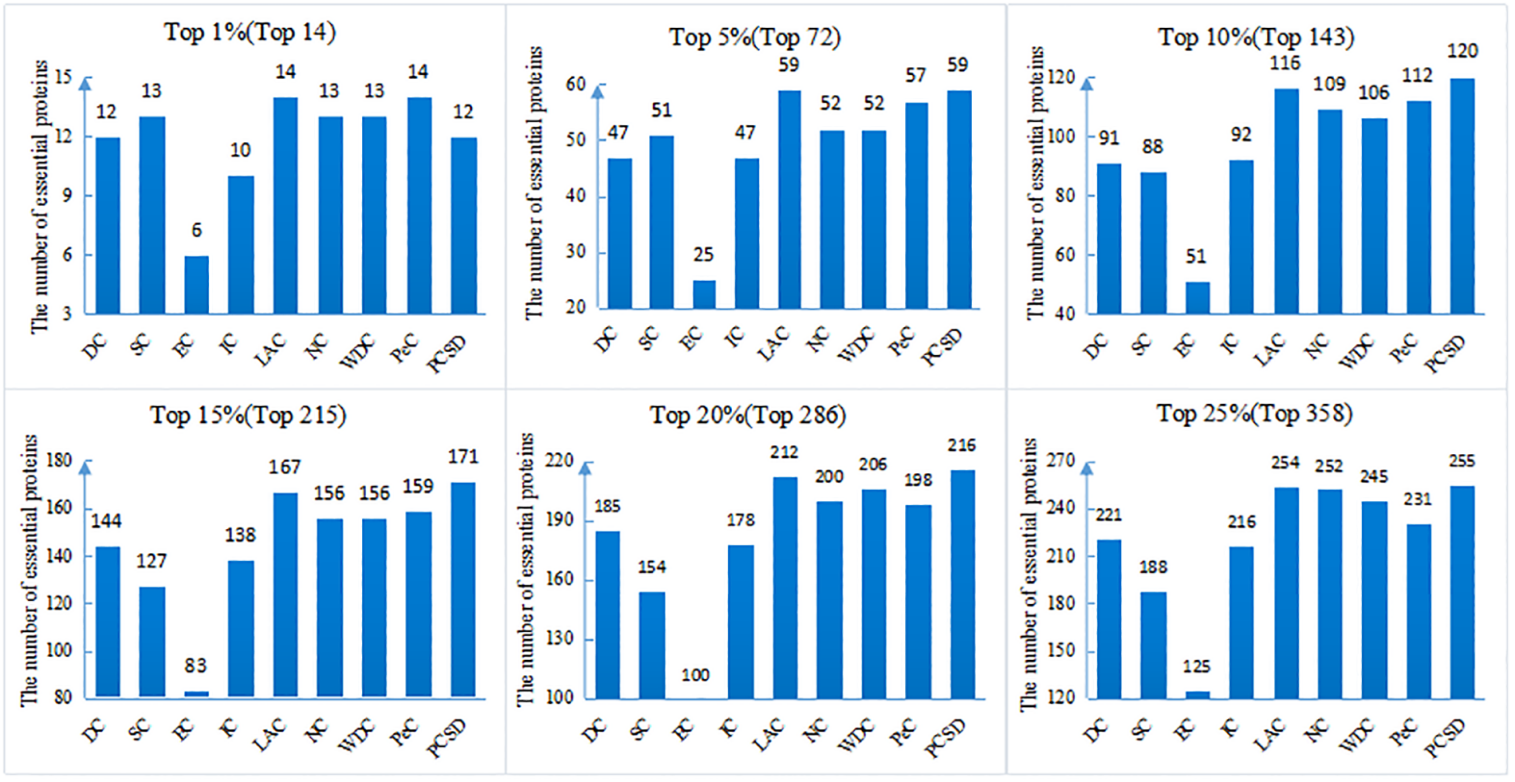
The number of true essential proteins predicted by PCSD and other several methods on Gavin dataset.

In this section, we compare PCSD with other essential proteins prediction methods (DC, SC, EC, IC, LAC, NC, WDC, PeC, UDoNC and LBCC) using the four datasets described in the Experimental data section. As UDoNC needs protein domain data, for convenience, UDoNC is only applied on DIP PPI network as mentioned in their paper [18]. And LBCC is applied on DIP and MIPS datasets as mentioned in their paper [22]. First, proteins are ranked in descending order according to their scores calculated by each method. Then, the top 1, 5, 10, 15, 20, 25 percent of all proteins are selected as candidate essential proteins, and finally, the number of true essential proteins in these essential protein candidates is determined according to gold standard dataset of known essential proteins. We visualize the proportion of essential proteins in top ranked proteins for all methods. The comparative results are shown in Figs 1–4. The method PCSD was conducted on four refined PPI networks and the other methods were conducted on original PPI networks.

For the DIP dataset shown in Fig 1, PCSD outperforms all the other ten methods from top 1% to 15% of ranked proteins, and LBCC has the best performance at top 20% and top 25%. Let us take the top 1% as an example, 45 essential proteins are correctly predicted by PCSD while 22, 24, 24, 24, 29, 32, 36, 39, 37and 37 for DC, SC, EC, IC, LAC, NC, WDC, PeC, UDoNC and LBCC, respectively.

For the Krogan dataset shown in Fig 2, PCSD achieves the best performance compared with other eight methods from top 1% to top 25% of ranked proteins. Let us take the top 25% of ranked proteins as an example, 351 essential proteins are correctly predicted by PCSD while 318, 272, 253, 314, 325, 323, 332 and 317 for DC, SC, EC, IC, LAC, NC, WDC and PeC, respectively.

For the MIPS dataset shown in Fig 3, LBCC obtains the best results from top 1% and 25% of ranked proteins. Except for LBCC, the performance of PCSD is obviously superior to that of the other eight methods at various proportions of top ranked proteins. And for the other eight compared methods, the largest number of true essential proteins identified are 16(PeC), 93(PeC), 185(PeC), 248(PeC), 312(WDC) and 374(WDC) at six percentages from top 1% to top 25%. By comparison, PCSD correctly predicted 33, 151, 272, 357, 426 and 475 essential proteins, and achieved more than 106, 62, 47, 43, 36 and 27 percent improvements, respectively.

For the Gavin dataset shown in Fig 4, compared with other eight methods, PCSD can identify more essential proteins at the 5%, 10%, 15%, 20% and 25% of top ranked proteins. At top 1% level, LAC and PeC correctly identified all 14 true essential proteins, the number of true essential proteins identified by PCSD is 12, which is near to the result obtained by LAC and PeC.

Thus, experiment results stated above indicate that PCSD can more effectively predict essential proteins than the other methods in most cases.

### Validation with jackknife methodology

**Fig 5:**
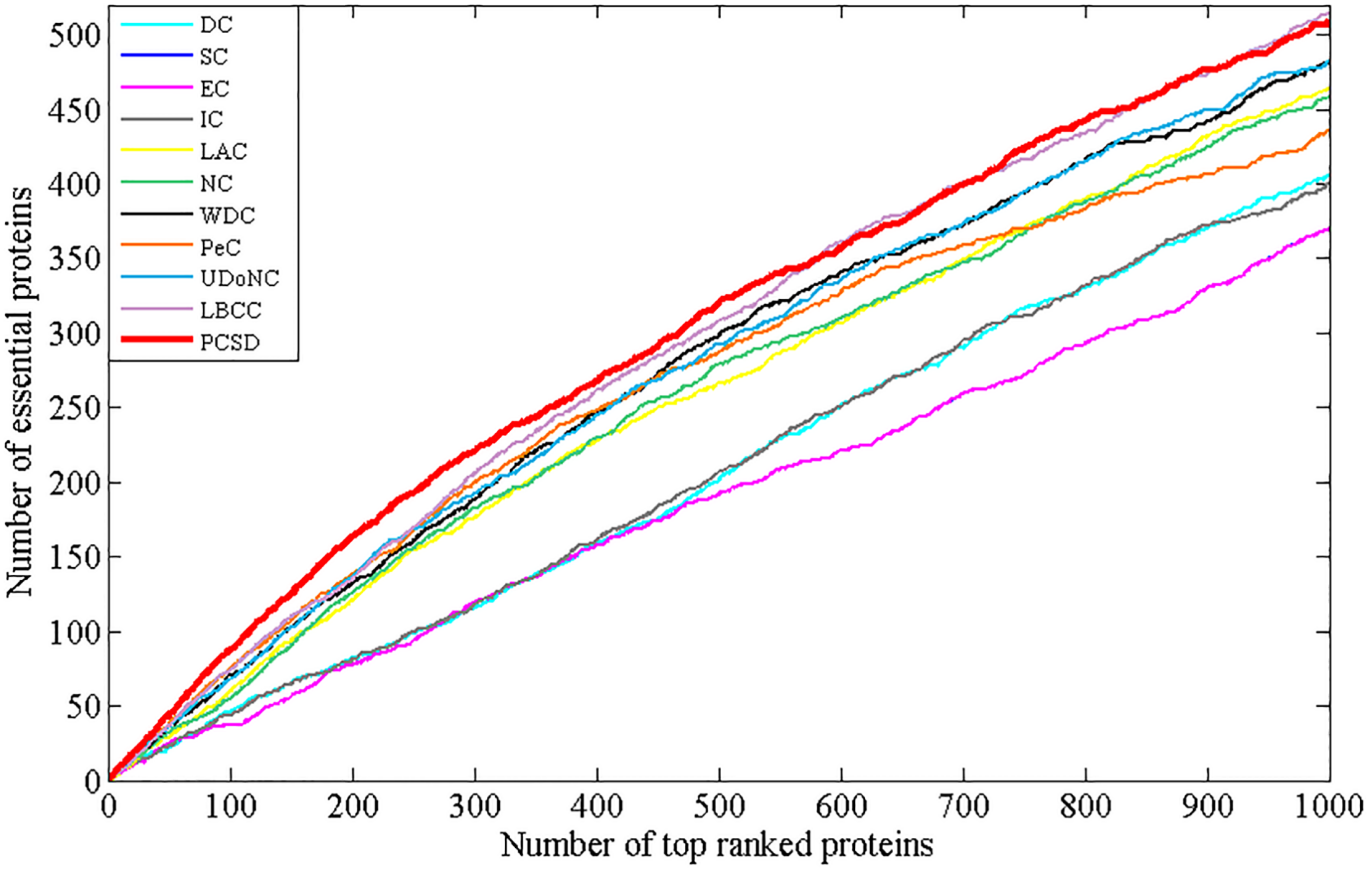
The jackknife curves of PCSD and other several methods for the DIP dataset.

**Fig 6:**
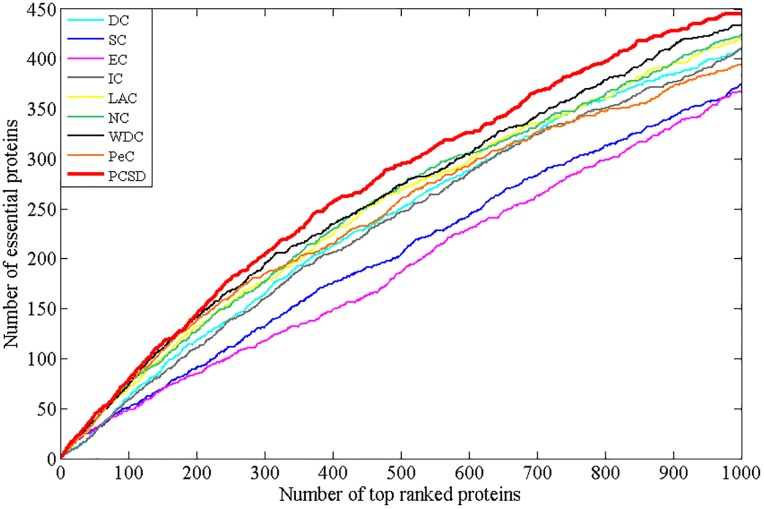
The jackknife curves of PCSD and other several methods for the Krogan dataset.

**Fig 7:**
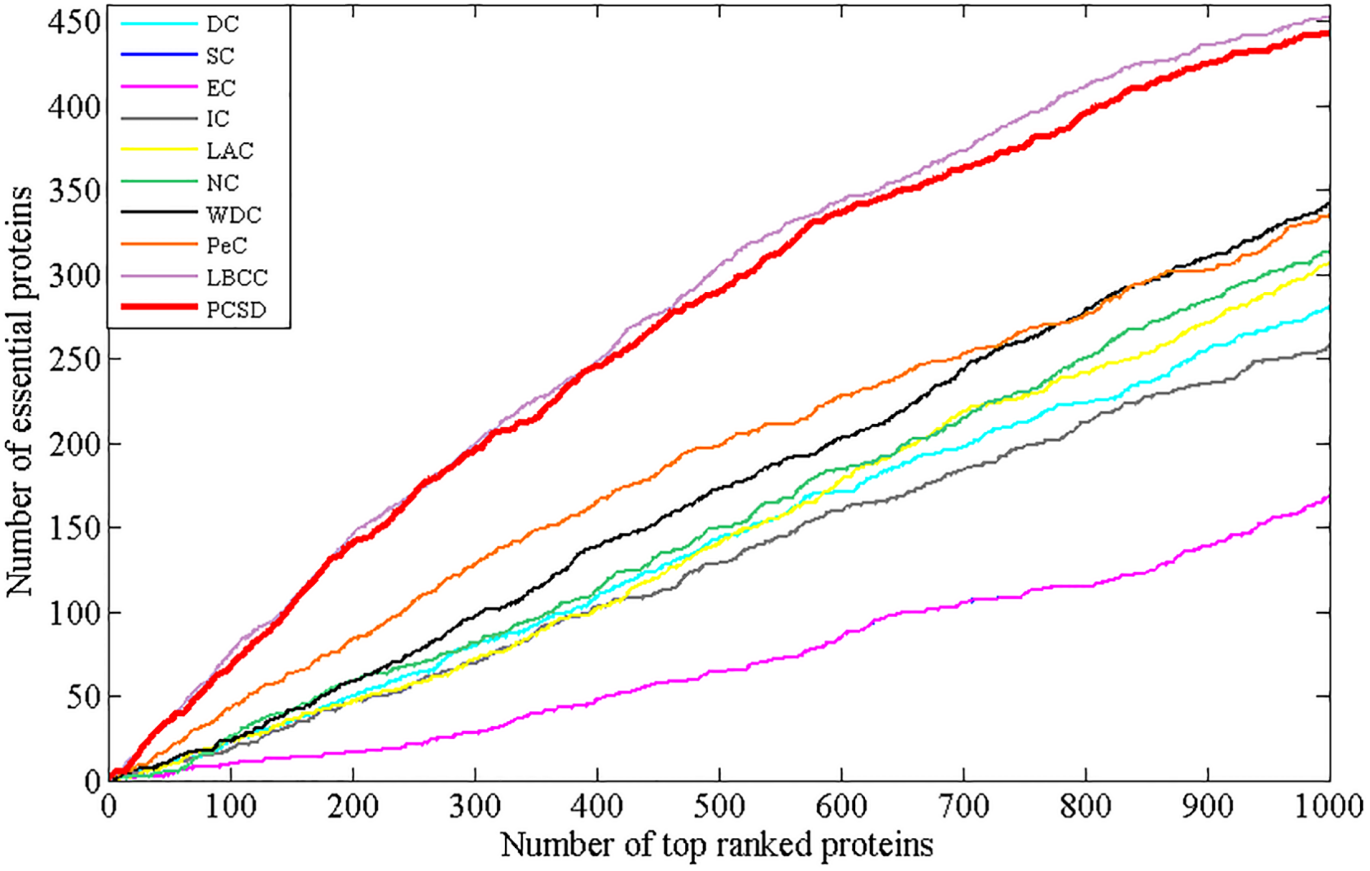
The jackknife curves of PCSD and other several methods for the MIPS dataset.

**Fig 8:**
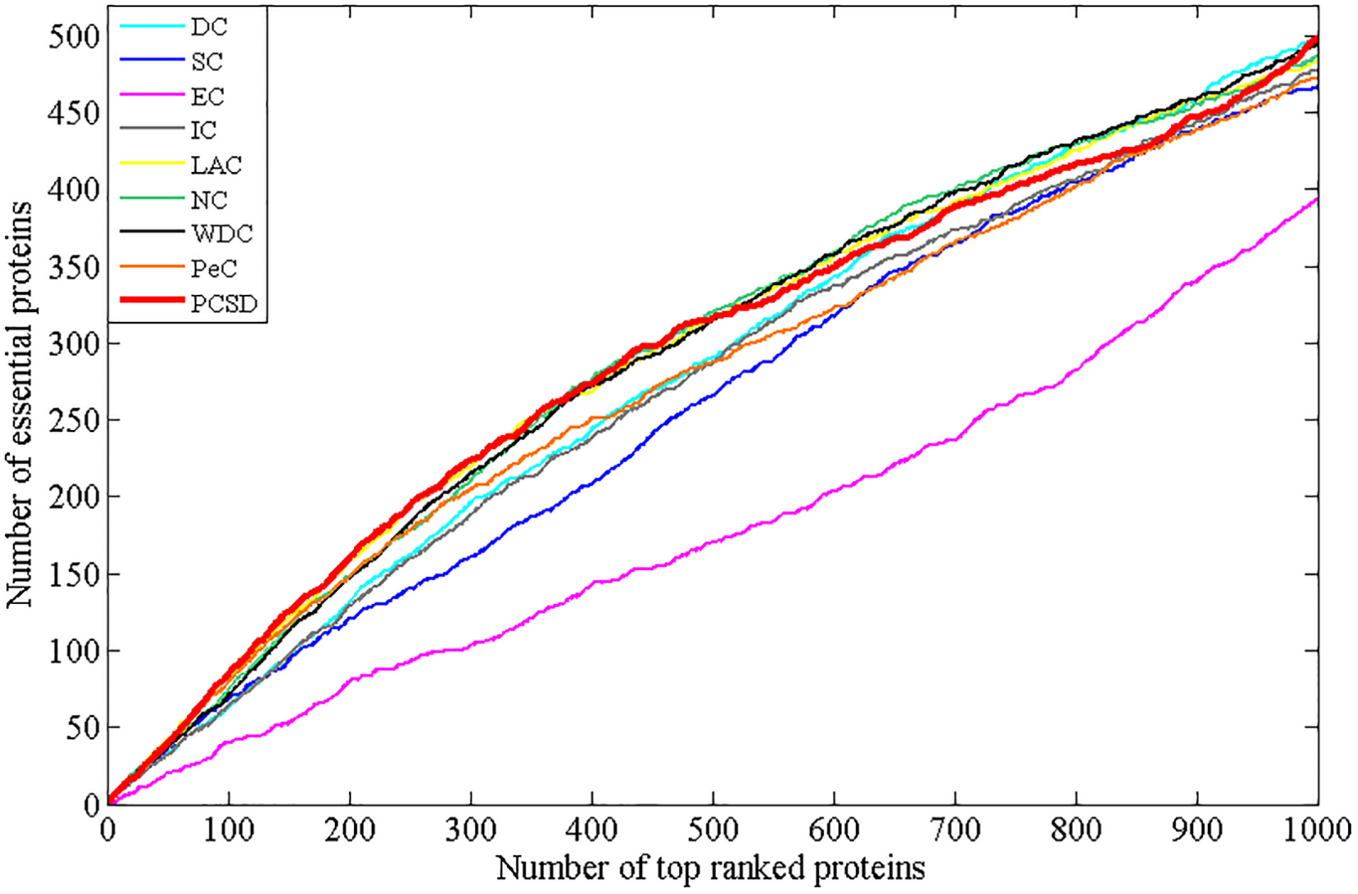
The jackknife curves of PCSD and other several methods for the Gavin dataset.

In this section, we employ the jackknife methodology to evaluate furtherly the performance of PCSD as well as other identification methods. The results are shown in Figs 5–8. The horizontal axis of the jackknife curves represents the proteins ranked based on scores of essentiality calculated by each method in descending order from left to right. We chose the top 1000 proteins for each dataset to analyze the performance of PCSD and other methods. The vertical axis of the jackknife curves represents the number of true essential proteins among the top *N* proteins, where *N* is the number along the horizontal axis. The Jackknife curve also reveal that our method PCSD has a better performance than other several methods.

### Validation with precision-recall curves

**Fig 9:**
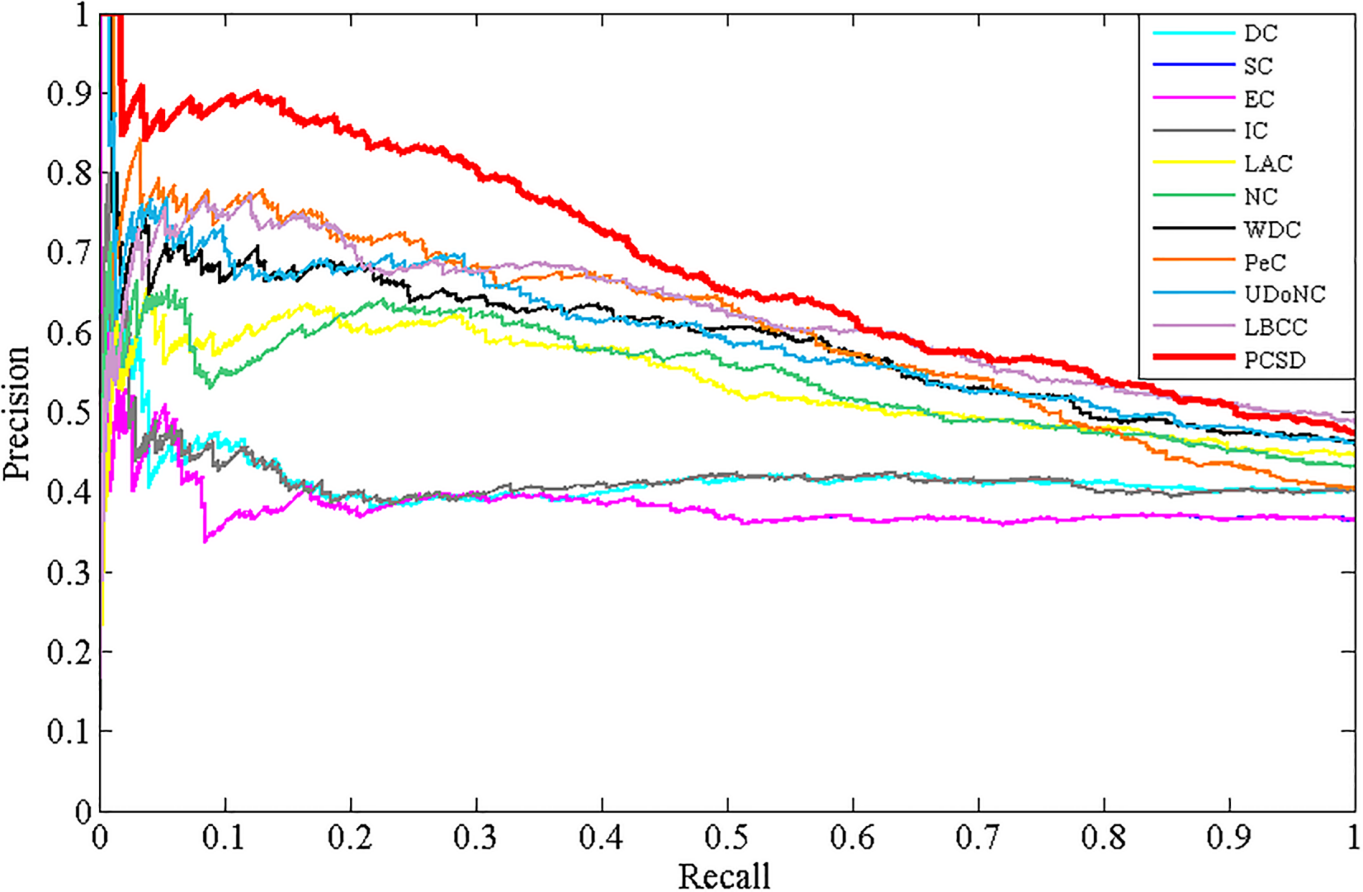
The PR curves of PCSD and other several methods for the DIP dataset.

**Fig 10:**
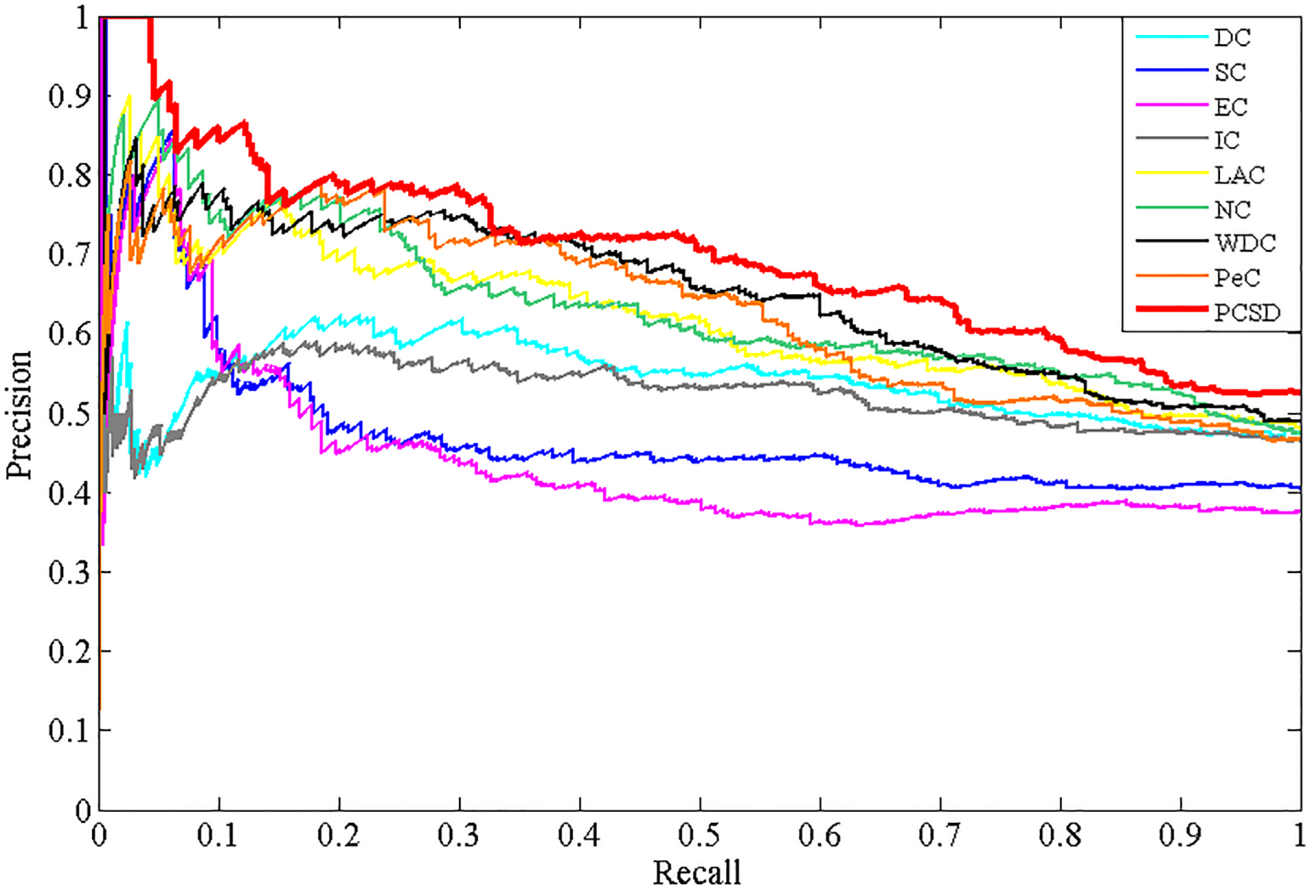
The PR curves of PCSD and other several methods for the Krogan dataset.

**Fig 11:**
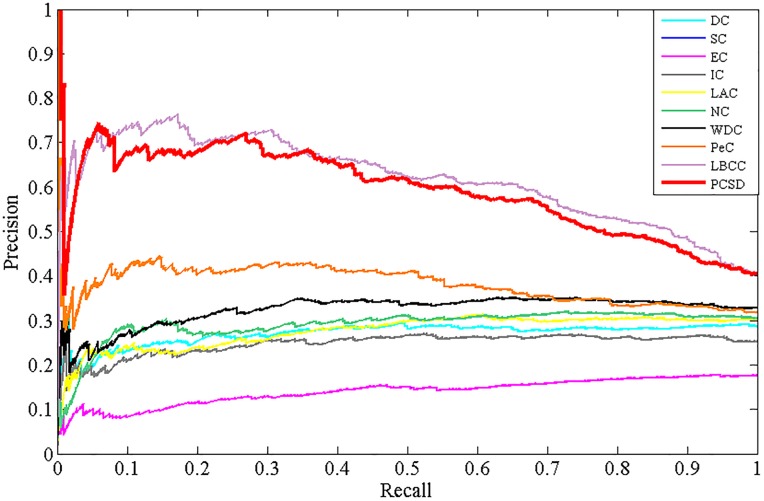
The PR curves of PCSD and other several methods for the MIPS dataset.

**Fig 12:**
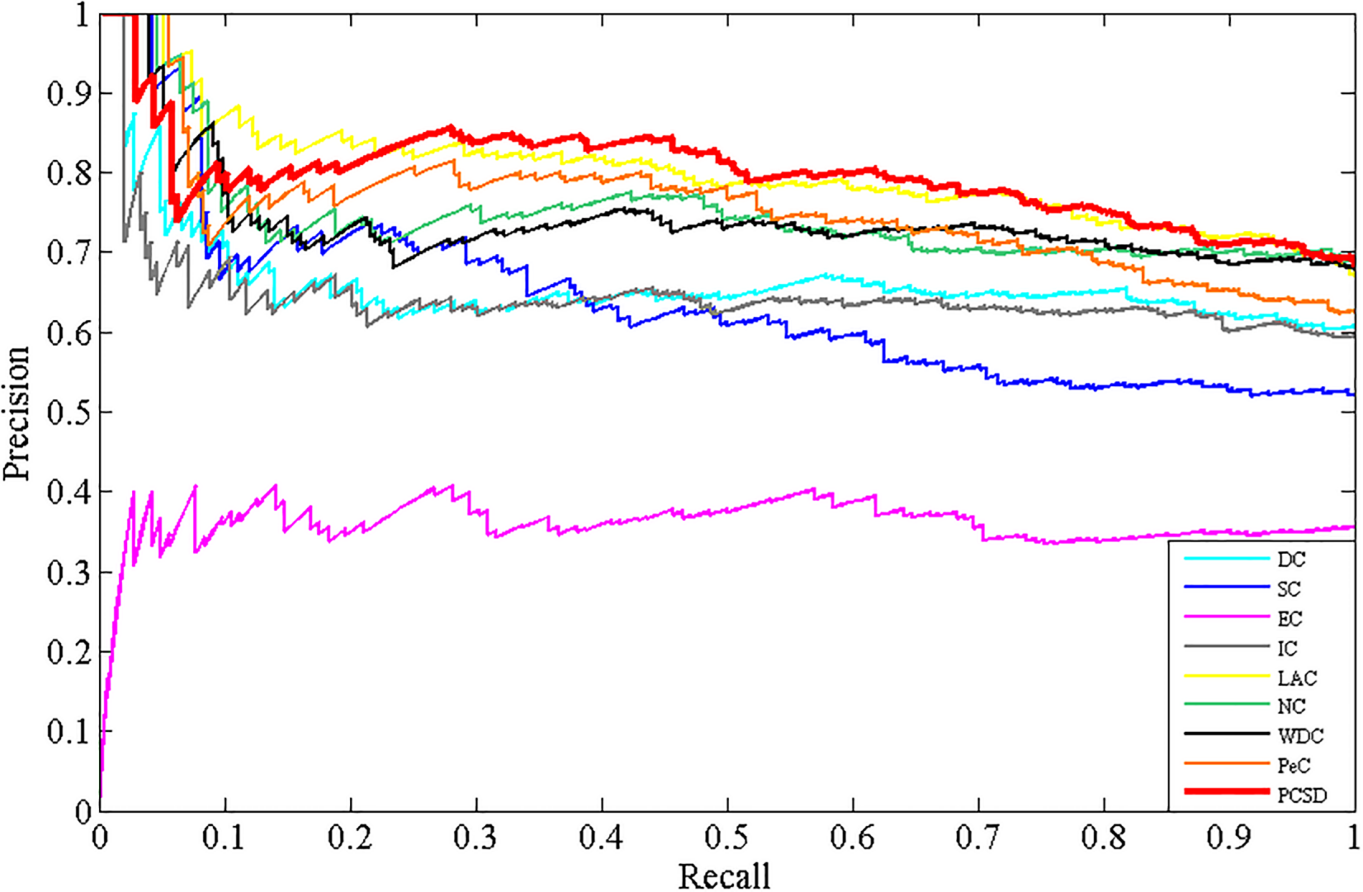
The PR curves of PCSD and other several methods for the Gavin dataset.

In addition, to assess the effectiveness of PCSD, we calculate the precision and recall of PCSD and other several methods, and plot the precision-recall cure for each method. Precision represents the proportion of predicted essential proteins that match the known ones. Recall represents the proportion of known essential proteins that are matched by predicted ones. They are defined as follows:

$$Precision=\frac{TP}{TP+FP}$$ (10)

$$Recall=\frac{TP}{TP+FN}$$ (11)

where *TP* is the number of true positives, which denotes essential proteins correctly identified as essential, *FP* is the number of false positives, which denotes non-essential proteins incorrectly predicted as essential and *FN* is the number of false negatives, which denotes essential proteins incorrectly predicted as non-essential. The results are shown as Figs 9–12, from which we can observe that compared with other methods, the PR curve of the new proposed method has an improvement on predicting essential proteins for all the four different datasets.

### The analysis of refining PPI networks

**Fig 13:**
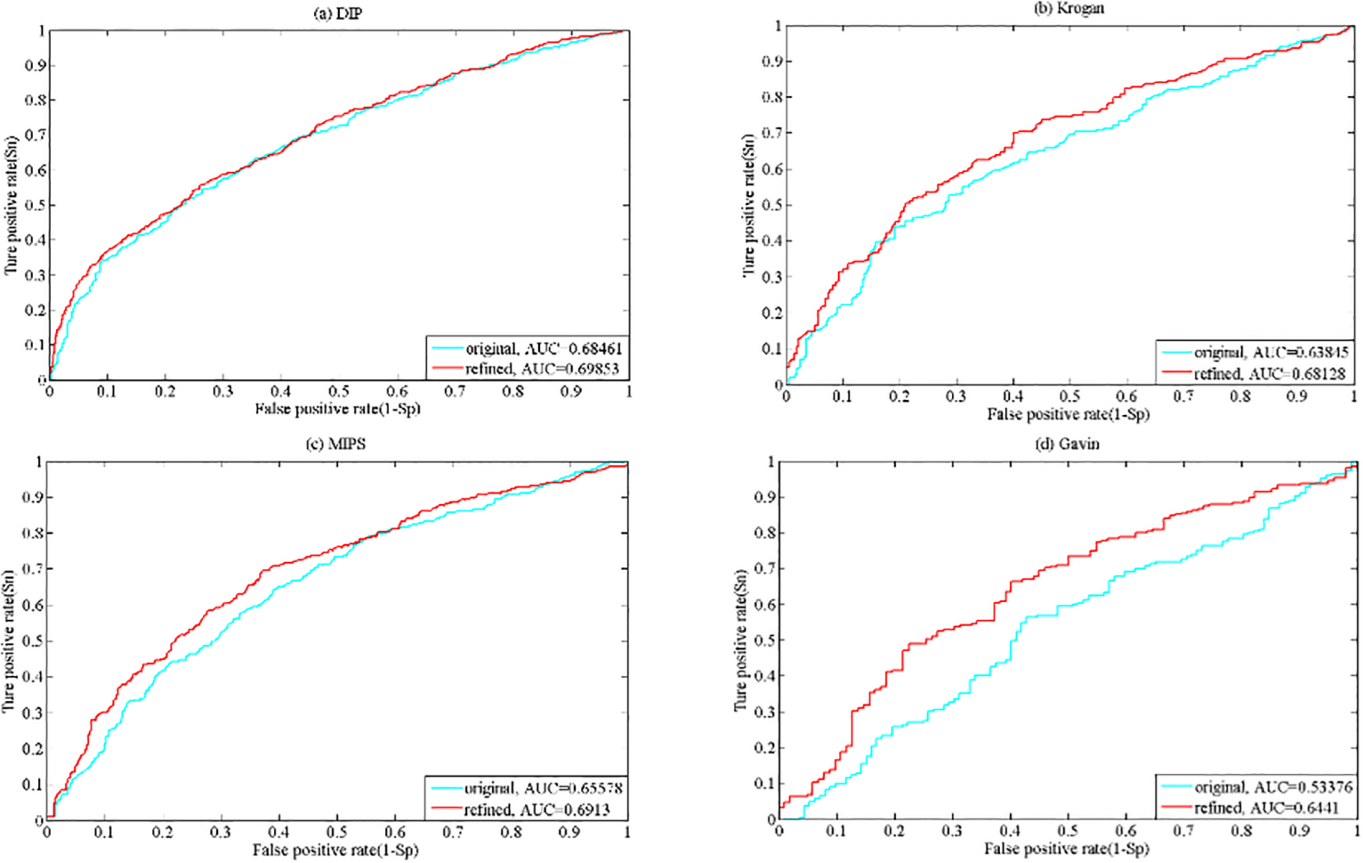
The ROC curves on original and refined PPI networks for (a) DIP dataset, (b) Kroan dataset, (c) MIPS dataset and (d) Gavin dataset.

In the PCSD method, to improve the prediction precision of essential proteins, refined PPI networks are constructed by deleting those unreliable protein-protein interactions in the first place. The numbers of edges of original and refined networks for four PPI datasets are shown in Table 2. In order to validate the effectiveness of refining PPI networks, we compare the prediction performance on original and refined PPI networks and plot The Receiver Operating Characteristics (ROC) curve, which is a good way of evaluating a classifier’s performance [48]. In an ROC curve, the horizontal axis represents the values of true positive rate (*TPR*) and vertical axis represents the values of false positive rate (*FPR*). They are defined as follows.

$$TPR=\frac{TP}{TP+FN}$$ (12)

$$FPR=\frac{FP}{FP+TN}$$ (13)

where the means of *TP*, *FP* and *FN* are the same with the ones in Eqs (10) and (11), and *TN* is the number of true negatives, which denotes non-essential proteins correctly predicted as non-essential. The area under the ROC curves (AUC) is used to measure the performance of predicting essential proteins on original and refined PPI networks, the larger the AUC value is, the better the prediction performance is. The ROC curves for four PPI datasets are shown in Fig 13, from which we can observe that the values of AUC on refined PPI networks are always higher than those on original PPI networks for four different datasets. The AUC are 0.68461 and 0.69853 for original and refined DIP PPI network, respectively, and there is a little improvement. However, the prediction performance on refined PPI network is obviously better compared with that on original PPI network for Krogan, MIPS and Gavin datasets. Therefore, it is effective to improve the essential proteins identification precision by refining the original PPI networks.

**Table 2:** The number of edges for original and refined PPI networks.

Dataset	The number of edges for original network	The number of edges for refined network	The number of edges deleted
**DIP**	24743	10715	14028
**Krogan**	7075	3381	3694
**MIPS**	12319	6937	5382
**Gavin**	6531	3653	2878

### The analysis of parameter α

In our method PCSD, the ranking scores of proteins compose of two parts: participation degree in complexes and subgraph density, which are adjusted by parameter α. We set the value of α ranges from 0 to 1. When α is assigned as 0, 0.1, 0.2, … 0.9 and 1, respectively, the prediction results of PCSD are presented in Table 3. When α = 0, only the subgraph density is considered, and when α = 1, only the participation degree in complexes is considered. From Table 3, we can see that when the value of α ranges from 0.5 to 1, the performance of PCSD is better. Because the performance of PCSD has slight difference when predicting the top 15%, 20% and 25% of top ranked proteins, we set the value as 0.8 for α to conduct experiments on four datasets in this study.

**Table 3:** The number of true essential proteins correctly identified by PCSD with different α.

dataset	α	Top 1%	Top 5%	Top 10%	Top 15%	Top 20%	Top 25%
**DIP**	0	17	67	108	159	217	285
	0.1	44	194	312	405	487	541
	0.2	45	197	321	413	500	557
	0.3	45	195	322	421	504	570
	0.4	45	197	321	427	508	573
	0.5	45	197	323	429	513	573
	0.6	45	197	323	429	515	577
	0.7	45	197	323	430	514	578
	0.8	45	196	323	430	514	579
	0.9	45	196	323	430	511	579
	1	45	196	323	431	511	571
**Krogan**	0	8	54	90	133	165	206
	0.1	23	90	169	246	294	342
	0.2	23	105	179	250	306	346
	0.3	23	104	188	248	304	345
	0.4	23	104	188	249	308	347
	0.5	23	104	188	254	310	346
	0.6	23	104	187	255	308	347
	0.7	24	104	188	256	307	349
	0.8	24	104	188	258	305	351
	0.9	24	104	188	257	304	354
	1	24	104	188	258	303	356
**MIPS**	0	26	95	169	230	297	372
	0.1	27	143	254	330	405	463
	0.2	34	153	267	351	408	474
	0.3	35	161	269	354	413	471
	0.4	33	158	272	357	419	472
	0.5	33	159	272	354	423	475
	0.6	33	154	276	356	424	475
	0.7	33	154	275	356	426	475
	0.8	33	151	272	357	426	475
	0.9	33	152	273	357	426	474
	1	33	152	272	358	425	457
**Gavin**	0	6	18	51	85	114	142
	0.1	12	53	106	152	198	237
	0.2	12	59	121	160	206	245
	0.3	12	59	120	172	215	247
	0.4	12	59	120	171	214	254
	0.5	12	59	120	171	215	256
	0.6	12	59	120	171	216	255
	0.7	12	59	120	171	216	256
	0.8	12	59	120	171	216	255
	0.9	12	59	120	171	216	255
	1	12	59	120	171	216	255

## Conclusions

Essential proteins play a crucial role in the viability and reproduction of living organisms, and the identification of essential proteins contribute to promoting the process of disease study and drug design. At present, there are many computational methods proposed to detect essential proteins. In our study, we have proposed a new essential proteins prediction method that integrates participation degree in protein complexes and subgraph density, named PCSD. First, we construct a refined PPI network (RPIN), then, we calculate the participation degree in complexes for each protein based on the weighted RPINs generated by Edge Clustering Coefficient (*ECC*) and Pearson Correlation Coefficient (*PCC*), which determines the topological properties and co-expression characteristics of proteins, respectively. In addition, we construct a subgraph for each protein within the second order of neighbors, and weight the interactions in the subgraph based on sharing GO annotations (*SG*) and sharing protein complexes (*SC*), then the subgraph density is measured. Experiment results have shown that the proposed PCSD method can make an improvement in predicting essential proteins. Furthermore, researches have suggested that there is a close relationship between essential proteins and causing disease gene, so we will focus on identifying and prioritizing disease-related genes by combing various data sources in future.

## Supporting information

S1 Excel Standard essential proteins data. (XLSX)

S2 Excel Gene expression data. (XLSX)

S3 Excel Protein complex data. (XLSX)

S4 Excel GO annotation data. (XLSX)

S1 Text Protein interaction data in original DIP dataset. (TXT)

S2 Text Protein interaction data in original Krogan dataset. (TXT)

S3 Text Protein interaction data in original MIPS dataset. (TXT)

S4 Text Protein interaction data in original Gavin dataset. (TXT)

S5 Text Protein interaction data in refined DIP dataset. (TXT)

S6 Text Protein interaction data in refined Krogan dataset. (TXT)

S7 Text Protein interaction data in refined MIPS dataset. (TXT)

S8 Text Protein interaction data in refined Gavin dataset. (TXT)
